# Supplementary material for: Preferences for cesarean section among pregnant women at a tertiary hospital in Ho Chi Minh City, Vietnam: Influencing factors and implications for prenatal care
Source: PLoS One. 2025 Oct 29;20(10):e0335082. doi: 10.1371/journal.pone.0335082 (PMC12571310; doi:10.1371/journal.pone.0335082)
Supplement: S1 Data — (ZIP) [file pone.0335082.s001.zip › Data dictionary.docx]

| **Variable name** | **Variable label** |
| --- | --- |
| **id** | id |
| **a1** | Age |
| **a2** | Residence group |
| **a3** | Ethic group |
| **a4** | Religion |
| **a5** | Academic level |
| **a6** | Height |
| **a7** | Weight |
| **a8** | Occupation |
| **a11** | Economic status |
| **a12** | Health insurance |
| **a15** | Antenatal class attendance |
| **a17** | Yoga class attendance |
| **b1** | Nulli-multiparous |
| **b2** | Previous Delivery |
| **b2b** | Parity |
| **b3** | Deep perineal laceration in previous delivery |
| **b4** | Infection of the perineal suture |
| **b5** | Complications in previous birth |
| **b6** | Labor lasting >24 hours in a previous delivery |
| **b7** | Obstetric pain relief in previous delivery |
| **b9** | Getting chronic diseases during pregnancy |
| **b11** | Anemia |
| **b12** | IVF |
| **b13** | Gestational age |
| **b14** | Estimated current fetal weight using ultrasound |
| **b15** | The fetus has problems |
| **b16** | Amount of amniotic fluid |
| **b17** | Number of child you plan to have |
| **b18** | Delivery mode preference in this delivery |
| **c1** | Healthy gestation |
| **c2** | Belief in your ability in giving vaginal birth |
| **c3** | Fear of labor pain |
| **c4** | Fear of episiotomy |
| **c7** | Fear of failure vaginal delivery |
| **c8** | Fear of complication of vaginal delivery |
| **c9** | Labor companionship expectation |
| **c12** | CS has a less pain than VD |
| **c13** | Safer for the mother |
| **c14** | Concern about postpartum sexual activities |
| **c15** | Allows better control of time of birth |
| **c16** | Date of birth affect to the family's life |
| **c17** | Choose date of birth |
| **c19** | Exposing to negative experience of other women |
| **c20** | Receiving the relatives's advice for CS |
| **c22** | Recommendation of healthcare provider for CS |
| **c24** | Safer for the baby |
| **d1** | Vaginal birth helps shorten hospital stays |
| **d2** | Blood loss in vaginal birth is usually less than a cesarean section |
| **d3** | Postpartum lochia drains better |
| **d4** | Increase your chances of successful breastfeeding |
| **d5** | Reduced risks associated with surgery |
| **d6** | Faster recovery, quick movement after giving birth |
| **d7** | Skin to skin with the baby immediately after birth |
| **d8** | Reduce the risk of complications during future pregnancies |
| **d9** | Normal birth costs less than cesarean birth |
| **d10** | The time to get pregnant again in vaginal birth is shorter than cesaream section |
| **d11** | Babies born vaginally are less likely to have symptoms of respiration failure |
| **d12** | Through vaginal birth baby can be touched to mother sooner that helps to develop |
| **d13** | Baby exposure to the mother's vaginal useful microbiota |
| **d14** | There is the possibility of an emergency cesarean section during labor |
| **d15** | Have possibility of getting instrumental birth assistance |
| **d17** | Have possibility of postpartum vulvar/vaginal pain |
| **d18** | Avoid pain during labor |
| **d19** | Avoid long/prolonged labor |
| **d20** | Reduce the risk of needing an emergency cesarean section or assisted birth |
| **d21** | Avoid episiotomy pain |
| **d23** | Risk of complications due to epidural anaesthesia |
| **d24** | Losing more blood than vaginall birth, affects the mother's health |
| **d25** | The hospital stay for a cesarean section is longer than a normal birth |
| **d26** | Slow recovery |
| **d27** | Pain extension after birth |
| **d28** | Reduced chances of breastfeeding after giving birth |
| **d29** | Increased risk of complications after surgery |
| **d30** | Increased risk for future pregnancies |
| **d31** | Surgical scars affects to appearance |
| **d32** | Risk of respiratory disorders for baby |
