# Supplementary material for: Preferences for cesarean section among pregnant women at a tertiary hospital in Ho Chi Minh City, Vietnam: Influencing factors and implications for prenatal care
Source: PLoS One. 2025 Oct 29;20(10):e0335082. doi: 10.1371/journal.pone.0335082 (PMC12571310; doi:10.1371/journal.pone.0335082)
Supplement: S1 Data — (ZIP) [file pone.0335082.s001.zip › Questionnaire.docx]

**Questionnaire**

**SECTION A: Personal Information**

| **No** | **Question** | **Answer** | **Code** | **Note** | **Definition** |
| --- | --- | --- | --- | --- | --- |
| A1 | Date of birth | ……………………. |  |  | Calculated by subtracting the year of birth from the year 2023 |
| A2 | Residence | HCM city  Others | 0  1 |  | Defined as the current place of residence of the pregnant woman |
| A3 | Ethic | Kinh  Hoa  Other (specific) | 1  2  3 |  | Defined based on asking the participants about their ethic as recorded on their identification card. |
| A4 | Religion | No  Yes | 0  1 |  | Defined based on asking the participants about their religion as recorded on their identification card. |
| A5 | Academic level | Primary school  Secondary School  High school  University | 1  2  3  4 |  | Defined based on the highest level of education that the participants had completed |
| A6 | Height (cm) | …………………. |  |  | Defined based on the current height of the participants as recorded in the medical record |
| A7 | Weight (kg) | ……………………………… |  |  | Defined based on the current weight of the participants as recorded in the medical record |
| A8 | Occupation | Housewife  Blue worker  Office worker  Entrepreneur  Freelancer | 1  2  3  4  5 |  | Defined as the occupation that provided the primary income for the participants |
| A9 | Economic status | < 20 millions VND  >20 millions VND | 1  2 |  | Defined as the total monthly income of the family, excluding the income of previous generations if they were living together in the same household. |
| A10 | Health Insurance | No  Yes | 0  1 |  | Defined as individuals who participated in health insurance, including either state or private health insurance. |
| A11 | Antenatal class attendance | No  Yes | 0  1 |  | Defined as individuals who had participated in prenatal care classes organized at the hospital |
| A12 | Yoga class attendance | No  Yes | 0  1 |  | Defined as individuals who had participated in yoga classes organized at the hospital. |

**SECTION B: Obstetric Factors**

| **No** | **Question** | **Answer** | **Code** | **Note** | **Definition** |
| --- | --- | --- | --- | --- | --- |
| **Obstetric prehictoric** | | | | |  |
| B1 | Parity | Nulliparous  Multiparous | 0  1 |  | Defined as the current number of children of the pregnant woman had. |
| B2 | Previous Delivery | Vaginal delivery  Cesarean section | 0  1 |  | Defined as the previous birth mode of the participants |
| B3 | Deep perineal laceration in previous delivery | No  Yes | 0  1 |  | Defined as individuals who had previously experienced a third- or fourth-degree perineal tear during previous deliveries |
| B4 | Infection of the perineal suture (swelling, heat, redness, pain) in previous delivery | No  Yes | 0  1 |  | Defined as individuals who had previously experienced infection of the perineal suture (swelling, heat, redness, pain) in previous deliveries |
| B5 | Complications in previous birth (PPH, baby suffocation) | No  Yes | 0  1 |  | Defined as individuals who had previously experienced postpartum hemorrhage or whose baby exhibited signs of asphyxia, shoulder dystocia, respiratory distress, or other issues requiring transfer to the Neonatal Care Unit for treatment in a previous deliveries |
| B6 | Labor lasting >24 hours in a previous delivery | No  Yes | 0  1 |  | Defined as individuals who experienced a prolonged labor lasting more than 24 hours during a previous delivery, calculated from the onset of labor signs such as bloody show or periodic abdominal pain until successful delivery |
| B7 | Obstetric pain relief in previous delivery | No  Yes | 0  1 |  | Defined as individuals who used medical obstetric pain relief in a previous deliveries |
| **Obstetric Status** | | | | |  |
| B9 | Getting chronic diseases during pregnancy | No  Yes | 0  1 |  | Defined as individuals who had experienced medical conditions during pregnancy or prior to it, such as hypertension, diabetes, asthma, heart disease, Hepatitis B, and other related conditions |
| B10 | Type of chronic diseases | High blood pressure  Diabetes  Asthma  Hearth diseases  Others (specific…………….) | 1  2  3  4  5 |  |  |
| B11 | Anemia (based on the lastest blood test, (Hb<10g/l)) | No  Yes | 0  1 |  | Defined as individuals who were diagnosed with anemia when hemoglobin (Hb) < 12 g/dL, hematocrit (Hct) < 37%, or red blood cell count (RBC) < 4 million/μL, based on test results |
| **Fetal status** | | | | |  |
| B12 | IVF/IUI | No  Yes | 0  1 |  | Defined as participants who had baby by IVF/IUI |
| B13 | Gestational age | ……………………….. |  |  | Gestational age was collected at an antenatal visit when pregnant women were interviewed |
| B14 | Estimate current fetal weight | ………………………. |  |  | Ultrasound estimated fetal weight was collected at an antenatal visit when pregnant women were interviewed |
| B15 | The fetus has problems (IUGR, heart, brain…) | No  Yes  (Specific………………….) | 0  1 |  | defined as when the fetus showed an ultrasound result with a percentile <10, severe growth restriction, or abnormalities of the heart, ventricles, or other organs |
| B16 | Amount of amniotic fluid | Normal  Oligohydramnios  polyhydramnios | 1  2  3 |  | defined as the amount of amniotic fluid during pregnancy based on the ultrasound results in this examination |
| **Plan for this delivery** | | | | |  |
| B17 | Number of child you plan to have | 1 child  >2 children | 1  2 |  | Number of children participants plan to have |
| B18 | Delivery mode preference in this delivery | Vaginal delivery  Cesearean section | 0  1 |  | referred to the participant’s self-reported preferred birth method (vaginal or cesarean birth) in the absence of medical indications or institutional constraints |

**Section C: Attitude, Expectation of Pregnant women**

| **No** | **Question** | **Answer** | **Code** | **Note** | **Definition** |
| --- | --- | --- | --- | --- | --- |
| C1 | Healthy gestation | No  Yes | 0  1 |  | **Low-risk gestation** as pregnancy without any reported discomforts, high-risk medical conditions, or complications affecting the mother or fetus up to the time of the interview |
| C2 | Belief in your ability in giving vaginal birth | No  Yes | 0  1 |  | defined as individuals who had the thoughts and belief that they were capable of achieving a successful vaginal birth |
| C3 | Fear of labor pain | No  Yes | 0  1 |  | defined as individuals who answered that they were afraid of labor pain |
| C4 | Fear of episiotomy | No  Yes | 0  1 |  | defined as individuals who answered that they were afraid of episiotomy in vaginal birth |
| C7 | Fear of failure vaginal delivery | No  Yes | 0  1 |  | defined as individuals who answered that they were afraid of failure vaginal delivery |
| C8 | Fear of complication of vaginal delivery | No  Yes | 0  1 |  | defined as individuals who answered that they were afraid of complication of vaginal delivery |
| C9 | Labor companionship expectation | No  Yes | 0  1 |  | defined as individuals who wished to have a companion present during labor |
| C12 | CS has a less pain than VD | No  Yes | 0  1 |  | defined as individuals who believed that CS was less painful than vaginal birth |
| C13 | Safer for the mother | No  Yes | 0  1 |  | defined as individuals who believed that CS was safer for the mother |
| C14 | Concern about postpartum sexual activities | No  Yes | 0  1 |  | defined as individuals who responded that they were concerned vaginal birth might affect sexual activities |
| C15 | Allows better control of time of birth | No  Yes | 0  1 |  | defined as individuals who responded that CS allowed better control of time of birth |
| C16 | Date of birth affect to the family’s life | No  Yes | 0  1 |  | defined as individuals who believed that date of birth affected to the family’s life |
| C17 | Choose date of birth | No  Yes | 0  1 | If answer “0” -> C18 | If answer “0” -> C18  defined as individuals or their family who chose date of giving birth |
| C18 | Who choose date of birth | grandpa/grandma  uncle/aunt  father/mother  wife/husband | 1  2  3  4 |  |  |
| C19 | Exposing to negative experience of other women | No  Yes | 0  1 |  | defined as individuals who had been exposed to negative experiences with vaginal birth, either directly or indirectly |
| C20 | Receiving the relatives’s advice for CS | No  Yes | 0  1 | If answer “0” -> C22 | If answer “0” -> C22  defined as individuals who had been advised by relatives, friends, or others to undergo CS |
| C21 | Who give advise for CS? (If any) | Husband’s family  Pregnant women’s family  Husband  Acquaintance | 1  2  3  4 |  |  |
| C22 | Recommendation of healthcare provider for CS | No  Yes | 0  1 |  | defined as individuals who had been advised by healthcare professionals to undergo CS |
| C23 | Smarter for baby | No  Yes | 0  1 |  | defined as individuals who believed that CS was smarter for the baby |
| C24 | Safer for the baby | No  Yes | 0  1 |  | defined as individuals who believed that CS was safer for the mother |

**Section D: Knowledge of the benefits and drawbacks of mode of birth**

| **No** | **Question** | **Answer** | **Code** | **Note** | **Definition** |
| --- | --- | --- | --- | --- | --- |
| **Vaginal delivery** | | | | |  |
| **Benefits** | | | | |  |
| D1 | Vaginal birth helps shorten hospital stays | No  Yes | 0  1 |  | Defined as participants who answered that VD helps shorten hospital stays |
| D2 | Blood loss in vaginal birth is usually less than a cesarean section | No  Yes | 0  1 |  | Defined as participants who answered that blood loss in vaginal birth is usually less than a cesarean section |
| D3 | Postpartum lochia drains better | No  Yes | 0  1 |  |  |
| D4 | Increase your chances of successful breastfeeding | No  Yes | 0  1 |  | Defined as participants who answered that VD increased their chances of successful breastfeeding |
| D5 | Reduced risks associated with surgery | No  Yes | 0  1 |  | Defined as participants who answered that VD reduced risks associated with surgery |
| D6 | Faster recovery, quick movement after giving birth | No  Yes | 0  1 |  | Defined as participants who answered that VD will be daster recovery, quick movement after giving birth |
| D7 | Skin to skin with the baby immediately after birth | No  Yes | 0  1 |  | Defined as participants who answered that VD helped mother skin to skin with the baby immediately after birth |
| D8 | Reduce the risk of complications during future pregnancies (Uterine rupture, placental abruption, placenta previa, placenta accreta, damage to organs near the surgical area…) | No  Yes | 0  1 |  | Defined as participants who answered that VD reduced the risk of complications during future pregnancies (Uterine rupture, placental abruption, placenta previa, placenta accreta, damage to organs near the surgical area…) |
| D9 | Normal birth costs less than cesarean birth | No  Yes | 0  1 |  | Defined as participants who answered that VD cost less than CS |
| D10 | The time to get pregnant again in vaginal birth is shorter than cesaream section | No  Yes | 0  1 |  | Defined as participants who answered that the time to get pregnant again in VD was shorter than CS |
| D11 | Babies born vaginally are less likely to have symptoms of respiration failure, and their lungs function is better | No  Yes | 0  1 |  | Defined as participants who answered that babies born vaginally were less likely to have symptoms of respiration failure, and their lungs function was better |
| D12 | Through vaginal birth baby can be touched to mother sooner that helps to develop baby’s emotion | No  Yes | 0  1 |  | Defined as participants who answered that VD helped to develop baby’s emotion through touching their mother sooner |
| D13 | Baby exposure to the mother’s vaginal useful microbiota helps to improve the baby’s intestinal microbiota. | No  Yes | 0  1 |  | Defined as participants who answered that VD helped baby exposure to the mother’s vaginal useful microbiota and improve the baby’s intestinal microbiota. |
| **Drawbacks** | | | | |  |
| D14 | There is the possibility of an emergency cesarean section during labor. | No  Yes | 0  1 |  | Defined as participants who answered that there was the possibility of an emergency cesarean section during labor |
| D15 | Have possibility of getting instrumental birth assistance | No  Yes | 0  1 |  | Defined as participants who answered that they had possibility of getting instrumental birth assistance |
| D16 | Have possibility of postpartum vulvar/vaginal pain | No  Yes | 0  1 |  | Defined as participants who answered that they had possibility of postpartum vulvar/vaginal pain |
| **Cesearean section** | | | | |  |
| **Benefits** | | | | |  |
| D17 | Avoid pain during labor | No  Yes | 0  1 |  | Defined as participants who answered that CS helped mother to avoid pain during labor |
| D18 | Avoid long/prolonged labor | No  Yes | 0  1 |  | Defined as participants who answered that CS helped mother to avoid long/prolonged labor |
| D19 | Reduce the risk of needing an emergency cesarean section or assisted birth | No  Yes | 0  1 |  | Defined as participants who answered that CS helped mother to reduce the risk of needing an emergency cesarean section or assisted birth |
| D20 | Avoid episiotomy pain | No  Yes | 0  1 |  | Defined as participants who answered that CS helped mother to avoid episiotomy pain |
| **Drawbacks** | | | | |  |
| D21 | Risk of complications due to epidural anaesthesia (Anesthesia, drug allergy, anaphylactic shock, hypotension) | No  Yes | 0  1 |  | Defined as participants who answered that CS increased risk of complications due to epidural anaesthesia |
| D22 | Losing more blook than vaginall birth, affects the mother’s health | No  Yes | 0  1 |  | Defined as participants who answered that CS would be caused of losing more blood than vaginal birth, affects the mother’s health |
| D23 | The hospital stay for a cesarean section is longer than a normal birth | No  Yes | 0  1 |  | Defined as participants who answered that the hospital stay for a CS was longer than a VD |
| D24 | Slow recovery, need more help in taking care of yourself and your baby | No  Yes | 0  1 |  | Defined as participants who answered that CS would be caused of slow recovery |
| D25 | Pain extension after birth | No  Yes | 0  1 |  | Defined as participants who answered that CS would be caused of pain extension after birth |
| D26 | Reduced chances of breastfeeding after giving birth | No  Yes | 0  1 |  | Defined as participants who answered that CS reduced their chances of breastfeeding after giving birth |
| D27 | Increased risk of complications after surgery (hematoma, intestinal paralysis…) | No  Yes | 0  1 |  | Defined as participants who answered that CS increased risk of complications after surgery |
| D28 | Increased risk for future pregnancies (uterine rupture, placental abruption, placenta previa, cesarean scarectopic pregnancy…) | No  Yes | 0  1 |  | Defined as participants who answered that CS increased risk for future pregnancies |
| D29 | Surgical scars affects to appearance | No  Yes | 0  1 |  | Defined as participants who answered that surgical scars of CS affected their appearance |
| D30 | Risk of respiratory disorders for baby | No  Yes | 0  1 |  | Defined as participants who answered that CS increased risk of respiratory disorders for baby |
