# Supplementary material for: Preferences for cesarean section among pregnant women at a tertiary hospital in Ho Chi Minh City, Vietnam: Influencing factors and implications for prenatal care
Source: PLoS One. 2025 Oct 29;20(10):e0335082. doi: 10.1371/journal.pone.0335082 (PMC12571310; doi:10.1371/journal.pone.0335082)
Supplement: Attachment — Submitted filename: Response to Reviewers.docx [file pone.0335082.s003.docx]

Dear Editor and Reviewers,

Thank you for providing thoughtful and valuable feedback on the manuscript. We have responded to the reviewer’s comments below and have changed the text accordingly.

We hope we have satisfactorily addressed all comments and look forward to hearing from you about this submission.

**Reviewers' comments:**

**Reviewer's Responses to Questions**

***Comments to the Author***

***1. Is the manuscript technically sound, and do the data support the conclusions?***

*The manuscript must describe a technically sound piece of scientific research with data that supports the conclusions. Experiments must have been conducted rigorously, with appropriate controls, replication, and sample sizes. The conclusions must be drawn appropriately based on the data presented.*

***Reviewer #1:*** *Yes*

**Response**: Thank you.

***Reviewer #2:*** *Partly*

**Response**: Thank you for your valuable feedback. We have revised the manuscript based on all the comments from the reviewers.

***2. Has the statistical analysis been performed appropriately and rigorously?***

***Reviewer #1:*** *No*

***Reviewer #2:*** *No*

**Response**: Thank you for your feedback regarding the statistical analysis. We acknowledge the concerns raised and have carefully reviewed and revised our statistical approach. Specifically, we have clarified the statistical methods used, ensured appropriate selection of tests, and provided additional justification for sample size determination and variable selection. We have also included more detailed explanations of our regression models and addressed potential confounding factors to improve the rigor of our analysis.

***3. Have the authors made all data underlying the findings in their manuscript fully available?***

*The PLOS Data policy requires authors to make all data underlying the findings described in their manuscript fully available without restriction, with rare exception (please refer to the Data Availability Statement in the manuscript PDF file). The data should be provided as part of the manuscript or its supporting information, or deposited to a public repository. For example, in addition to summary statistics, the data points behind means, medians and variance measures should be available. If there are restrictions on publicly sharing data—e.g. participant privacy or use of data from a third party—those must be specified.*

***Reviewer #1:*** *No*

**Response**: Thank you for your feedback. We acknowledge the importance of making all data fully available per the PLOS Data Policy. In response, we have included all relevant data as supplementary material and deposited the dataset in a publicly accessible repository, ensuring transparency and compliance with journal requirements. We have also updated the Data Availability Statement in the manuscript to reflect these changes.

***Reviewer #2:*** *Yes*

**Response**: Thank you.

***4. Is the manuscript presented in an intelligible fashion and written in standard English?***

*PLOS ONE does not copyedit accepted manuscripts, so the language in submitted articles must be clear, correct, and unambiguous. Any typographical or grammatical errors should be corrected at revision, so please note any specific errors here.*

***Reviewer #1:*** *No*

**Response**: Thank you for your feedback regarding the clarity and language of our manuscript. We have thoroughly revised the manuscript to correct typographical and grammatical errors and have refined the writing to ensure clarity and conciseness. Additionally, the manuscript was professionally proofread to enhance readability and ensure it adheres to standard academic English.

***Reviewer #2:*** *Yes*

**Response**: Thank you.

***5. Review Comments to the Author***

*Please use the space provided to explain your answers to the questions above. You may also include additional comments for the author, including concerns about dual publication, research ethics, or publication ethics. (Please upload your review as an attachment if it exceeds 20,000 characters)*

**Reviewer #1**

*Dear authors,*

*I have read your article with high interest and I could find some points that deserves attention.*

*Title, for example, i suggest you to modify it since your sample doesn't represent Vietnamese Women. I could not find the regional origin of the participants of this study, but i'm pretty sure they don't represent all women population.*

**Response**: Thank you for your insightful feedback. We have revised the title to more accurately reflect the study population*:* *“Preferences for Cesarean Section Among Pregnant Women at a Tertiary Hospital in Ho Chi Minh City, Vietnam: Influencing Factors and Implications for Prenatal Care.”* **(Title, lines 1-2)**

*In the introduction this should also be clarified that you consider only women followed by a specific hospital.*

**Response**: Thank you for your suggestion. We have revised the manuscript, to state: *“Therefore, our study aims to assess the preferences of women receiving care at Hung Vuong Hospital, a major tertiary referral maternity hospital in Ho Chi Minh City, regarding delivery modes and analyze the influencing factors, particularly for non-medical purposes.”* (**Introduction, 4^th^ Paragraph**).

*Methods:*

*For example, in settings you should mention the reason for choosing this hospital, and it would be great if you could describe a little bit about the prenatal coverage in the country. Can the antenatal care be made in primary healthcare networks? How accessible is the antenatal care for the population? Do people living in rural zone have easily access to antenatal care? I was also wondering in what gestational age most women started the prenatal in your country. Is there any regional discrepancies in CS and in prenatal rates?*

**Response**: Thank you. We have clarified the reason for selecting Hung Vuong Hospital in the Methods section: *“We conducted a cross-sectional study from 19th April 2023 to 04th June 2023 at Hung Vuong Hospital, a tertiary maternity facility in Ho Chi Minh City, Vietnam that provides care for both low- and high-risk pregnancies. Hung Vuong Hospital handles approximately 30,000 – 40,000 deliveries annually. The CS rate at the hospital has been increasing over time, nearly 47.6%. Many expectant mothers choose Hung Vuong Hospital over district hospitals due to its highly specialized medical staff, advanced facilities, and perceived higher safety standards. Furthermore, as most Vietnamese families have only one or two children, women tend to prioritize delivering at hospitals they perceive as the safest option.”* (**Setting and Participants, 1^st^ paragraph**)

*Another point was about the multiple regression. Although you briefly describe it, I was not sure if you used stepwise,..., and what was the VIF considered. Additionally, I advice you to do the Wald-test to bring more robust results.*

**Response**: Thank you for your comment. In response to this and a similar comment from Reviewer #2, we have re-run the multiple regression analysis using a stepwise backward selection approach. We also assessed multicollinearity using the Variance Inflation Factor (VIF) and evaluated model fit. The VIF values for the final model ranged from 1.03 to 1.32, indicating no multicollinearity issues. Model fit was supported by a Pearson’s Chi-squared test (p = 0.696) and a Hosmer-Lemeshow test (p = 0.748), both suggesting that the model fits the data well. We also performed Wald tests to assess the significance of individual variables and reinforce the robustness of the results. In the revised manuscript, we have included:

“*A backward elimination stepwise approach was used in multiple logistic regression with the initial inclusion of all variables with a p-value below 0.05 in the univariate analyses. The Hosmer Lemeshow test was used to evaluate the goodness of fit of the final model. Multicollinearity was assessed using the Variance Inflation Factor (VIF), and variables with VIF values above 5 were considered indicative of potential multicollinearity. Wald tests were also performed to assess the significance of individual predictors in the final model. A significance level of p < 0.05 was considered statistically significant*” **(Data analysis)**

**“***The VIF values for the final model ranged from 1.03 to 1.32, indicating no multicollinearity issues. Model fit was supported by a Pearson’s Chi-squared test (p = 0.696) and a Hosmer-Lemeshow test (p = 0.748), both suggesting that the model fits the data well.*” **(Results, 3rd paragraph)**

*From where you did take the "estimated proportion of CS preferences of 18.8%"?*

**Response**: Thank you. The estimated 18.8% of cesarean section (CS) preferences was derived from previous studies on maternal birth preferences in a Provincial Hospital in southern Vietnam. We have now clarified the source of this estimate in the Methods section (**Setting and Participants, 2^nd^ paragraph**).

*About questionary, was it evaluated by specialists before pilot test? I really was questioning myself about the origin of these* *voluntaries.*

**Response**: Thank you. We have revised the manuscript to clarify this:

“*Knowledge about birth modes was evaluated based on 30 questions about the advantages and drawbacks of vaginal delivery and CS. Techinical aspects of the study including the use of the questionnaire were evaluated by the Council for Science and Technology of the study hospital, which was composed of obstetricians and gynecologists. A pilot study was then conducted among 30 pregnant women to test the questionnaire which resulted in a high level of internal reliability with a Cronbach’s alpha of 0.804*”. (**Procedures and measurements, 1^st^ paragraph**)

*I see you also use descriptive analysis (n and %), but none was told in methods.*

**Response**: Thank you. We have added these in the revised manuscript: *“Frequencies and percentages summarized categorical variables.”* in the Statistical analysis (**Statistical Analysis**).

*In discussion it would be great if you could discuss about public policies in your country regarding the CS combat. Is there any specific law?*

**Response**: Thank you. We have revised the discussion to state:

“*Vietnam has not established a specific law to regulate or restrict elective CS without medical indications. However, without proper control measures, the rising CS rates may cause increased maternal and neonatal health risks and place a more substantial burden on the healthcare system. Thus, understanding and managing factors that contribute to a high rate of CS is crucial for ensuring health and reducing complications for both mother and fetus. In our study, the CS rate was relatively high and was associated with various factors. These associated factors included healthcare practitioners’ recommendations, the desire for better control over the timing of birth, advice from relatives, their exposure to negative childbirth experiences of others, and concerns about postpartum sexual activities.*” **(Discussion, 1^st^ paragraph)**

*Is there any public policy towards women's health?*

**Response**: We have revised the manuscript to include:

“*In Vietnam, the National Strategy for the Protection, Care, and Improvement of People's Health for the Period up to 2030, with a Vision to 2045, aims to develop a modern and internationally integrated healthcare system, enhance the quality of medical services, and achieve universal health coverage, with a particular focus on maternal and reproductive health. Additionally, the National Guidelines on Reproductive Health Services provide comprehensive guidance on reproductive healthcare, covering maternal care before, during, and after childbirth, family planning, and the prevention of sexually transmitted infections (STIs).*” **(Discussion, 2^nd^ paragraph)**

*What about cultural aspects? Because of that the origin of the participants is really important.*

**Response**: We ave revised the manuscript, to include:

***“****In Vietnam, folk beliefs suggest that a child’s destiny and future are influenced by their birth time and date. Considerable families opt for elective cesarean sections to ensure their baby is born during an auspicious hour, often based on feng shui advice. This practice has significantly contributed to the rising cesarean section rates, particularly in urban areas like Ho Chi Minh City and Hanoi”* **(Discussion, 3^rd^ paragraph)**

*In the study limitation should be included that your sample doesn't represent the national women population.*

**Response**: Thank you. We have included this in the study limitation:

“*this study was conducted at a single hospital, which may limit the generalizability of the findings to other regions or healthcare settings in Vietnam*” (**Discussion, 5^th^ paragraph)**

*Minor point: The wording of the text needs to be revised.*

**Response**: Thank you. We have thoroughly revised the manuscript to correct typographical and grammatical errors and have refined the writing to ensure clarity and conciseness. Additionally, the manuscript was professionally proofread to enhance readability and ensure it adheres to standard academic English.

***Reviewer #2***

*Thank you for the opportunity to review this paper which is related to the important issue of increasing CS rates in many countries.*

*This was a cross-sectional study of 599 pregnant women who received care at a single hospital in Ho Chi Minh City, Vietnam in 2023 to assess their preferences for birth mode.*

*They found 27% of women would prefer a CS with the main reasons including health practitioner recommendations and as well as different non-medical reasons.*

*Disclosure statements*

*Ethics statement is incomplete. Please indicate the type of consent obtained from the participant (as per Methods section).*

**Response**: Thank you. We included the type of consent obtained from the participants as this sentence: *“Written informed consent was obtained from all participants before their participation in the study.”* (**Ethical Approval**)

*General: I suggest using ‘birth mode’ instead of ‘delivery mode’ throughout the paper as this is more empowering for women.*

**Response**: Thank you. In the revised manuscript, we have replaced “delivery mode” with “birth mode.”

*Abstract: please define all abbreviations on first use (e.g. Odds ratio, confidence interval)*

**Response**: Thank you. We have clarified all the abbreviations in the revised manuscript.

*Introduction*

*Please categorize the potential risks of CS into maternal and fetal/infant risks. I also suggest adding the implications for later pregnancies (recurrent CS, uterine rupture).*

**Response**: Thank you. We have revised the manuscript, to state:

“*While CS can be life-saving in emergencies, its overuse without clear medical indications raises concerns about unnecessary risks for mothers and infants. These risks, which include increased chances of infection, more extended recovery periods, various subsequent pregnancy concerns, including placenta previa, uterine rupture, and stillbirth, recurrent CS and potential complications, such as respiratory issues, underscore the importance of understanding and using CS in clinical settings*” (**Introduction, 1^st^ paragraph**)

*Methods: Please add more details about the recruitment. Were all women who met the inclusion criteria or just a sample (random, convenience)? What was the participation fraction? These could also be added to Figure 1.*

**Response**: Thank you. In the revised anuscript, we have now stated:

“*Among the 661 pregnant women approached and recruited using a convenience sampling technique, 599 agreed to participate and completed the study, resulting in a participation rate of 90.6% (599/661).*” **(Setting and participants, 2^nd^ paragraph)**

*I suggest including the data collection instrument as supplementary material. Please describe how the birth characteristics (e.g. birth weight, gestation) were obtained. Were information about parity and birth mode for previous births obtained from the mother or her medical records? While this is mentioned in limitations, it should be described in methods first.*

*Please define ‘healthy gestation’ (item in Figure 2).*

**Response**: Thank you. We have added the questionaire of the study in the supplementary appendix. The birth characteristics (e.g. birth weight, gestation) were obtained from the examination record on the interview day when prenant women did prenatal care **(Procedures and measurements)**.

“Healthy gestation”: A pregnancy in which there are no discomforts or high risk concerns for either the mother or the fetus, and the mother feels well during the whole gestation

*Statistical Methods.*

*Please describe in more detail which variables were tested in the multivariable logistic models and list those included in the final models ( and list in a footnote in Figure 2).*

*I suggest doing sensitivity analyses by parity to compare the views of nulliparous women to multiparous women, and, among multiparous women compare women who had a previous CS to those who did not. This would allow the authors to better compare their findings to previous literature.*

**Response**: Thank you for the suggestion. We appreciate the idea of conducting sensitivity analyses by parity and history of cesarean section (CS), as it could help compare our findings with previous literature. However, both parity and previous CS were already included an independent variables in our final multiple regression model and was identified as significant predictors. Therefore, we believe the model already accounts for this factor, and additional subgroup analyses may not provide further meaningful insights beyond what is already captured.

Moreover, this variable had three groups: nulliparous women, multiparous women with previous vaginal birth, and multiparous women with previous CS. Doing the comparision between *previous CS and those who did not* would substantially reduce the number of observations in the final model and thereby affect the stability and power of the final model. For this reason, we opted to keep this variable in the model as a predictor (in the form of 3-category variable) rather than perform separate subgroup analyses.

*Results*

*Rather than giving mean maternal age, infant birth weight and gestation, I suggest showing these as categorical variables in Table 1. Was the reason for previous CS obtained from multiparous women who had had one?*

**Response**: Thank you. We have added maternal age, infant birth weight and gestation as categorical variables in Table 1 and have updated the result section, to state:

*“Among the participants, 18.7% of infants had a birth weight below 2500 grams, while the majority (81.3%) weighed 2500 grams or more at birth. Regarding gestational age, 80.0% of the women delivered between 36 and <38 weeks, and only 20.0% delivered at ≥39 weeks”.* **(Results, 1^st^ paragraph)**

Yes, the reason for previous CS was obtained from multiparous women who had had one.

*Table 1:*

*As the mean maternal age was 27 years and only a small percentage of women were ages over 40, it would be better to use different categorisation for maternal age (e.g <25, 25-29, 30-34 and 35 or more years). If the authors wish to use only 2 categories, a good choice is to split at the median value. The current categorisation may be the reason why no association was found between birth mode preference and maternal age.*

*Please also use more standard cut points for BMI. In my opinion, P values are not necessary in the table as the confidence intervals are sufficient. Please indicate the Reference group instead of OR = 1 ( as used in Figure 2).*

**Response**: Thank you. We have updated the manuscript to use 3 categories (e.g <25, 25-30,>30). Moreover, while we agree that confidence intervals provide valuable information about the precision and direction of the estimates, we have chosen to retain the p-values in the table to enhance clarity and facilitate interpretation for a broader audience, especially readers who may be more familiar with p-values as a measure of statistical significance.

We used 25 kg/m^2^ for cutting point of BMI according to WHO criteria for Asian obesity *(*[*https://www.sciencedirect.com/science/article/pii/S2667368124000585#bib11*](https://www.sciencedirect.com/science/article/pii/S2667368124000585#bib11)*).*

We used 39 weeks of gestation for cutting point according to the definition of term pregnancy of ACOG which recommended as full term of pregnancy (<https://www.acog.org/clinical/clinical-guidance/committee-opinion/articles/2013/11/definition-of-term-pregnancy>).

We have replaced OR=1 with Ref in Table 1.

*Discussion*

*As the authors mentioned, differences in health care systems may explain differences in both CS rates and women’s preferences. to give the reader more context, I suggest describing the health care system in Vietnam and particularly more details about Hung Vuong Hospital in the Introduction/Methods so the findings can be compared to similar settings. Please give details about the types of health care practitioners, whether Hung Vuong Hospital is a public or private hospital, level of care provided and how it compares to other birthing sites in Vietnam. Please also discuss the views of health providers regarding CS in Vietnam and describe the relevant guidelines for recommending a CS on medical grounds.*

**Response**: Thank you. We have added more details based on this recommendation.

*“We conducted a cross-sectional study from 19th April 2023 to 04th June 2023 at Hung Vuong Hospital, a tertiary maternity facility in Ho Chi Minh City, Vietnam, that provides care for both low- and high-risk pregnancies. Hung Vuong Hospital handles approximately 30,000 - 40,000 deliveries annually. The CS rate at the hospital has been increasing over time, nearly 47.6%.12 Many expectant mothers choose Hung Vuong Hospital over district hospitals due to its highly specialized medical staff, advanced facilities, and perceived higher safety standards. Furthermore, as most Vietnamese families have only one or two children, women tend to prioritize delivering at hospitals they perceive as the safest option.”* ***(Setting and participants, 1^st^ paragraph)***

“*Vietnam has not established a specific law to regulate or restrict elective CS without medical indications. However, without proper control measures, the rising CS rates may cause increased maternal and neonatal health risks and place a more substantial burden on the healthcare system. Thus, understanding and managing factors that contribute to a high rate of CS is crucial for ensuring health and reducing complications for both mother and fetus. In our study, the CS rate was relatively high and was associated with various factors. These associated factors included healthcare practitioners’ recommendations, the desire for better control over the timing of birth, advice from relatives, their exposure to negative childbirth experiences of others, and concerns about postpartum sexual activities.*” **(Discussion, 1^st^ paragraph)**

“*In Vietnam, the National Strategy for the Protection, Care, and Improvement of People's Health for the Period up to 2030, with a Vision to 2045, aims to develop a modern and internationally integrated healthcare system, enhance the quality of medical services, and achieve universal health coverage, with a particular focus on maternal and reproductive health. Additionally, the National Guidelines on Reproductive Health Services provide comprehensive guidance on reproductive healthcare, covering maternal care before, during, and after childbirth, family planning, and the prevention of sexually transmitted infections (STIs).*” **(Discussion, 2^nd^ paragraph)**

***“****In Vietnam, folk beliefs suggest that a child’s destiny and future are influenced by their birth time and date. Considerable families opt for elective cesarean sections to ensure their baby is born during an auspicious hour, often based on feng shui advice. This practice has significantly contributed to the rising cesarean section rates, particularly in urban areas like Ho Chi Minh City and Hanoi”* **(Discussion, 3^rd^ paragraph)**

***6. PLOS authors have the option to publish the peer review history of their article (what does this mean?). If published, this will include your full peer review and any attached files.***

*If you choose “no”, your identity will remain anonymous but your review may still be made public.*

*Do you want your identity to be public for this peer review? For information about this choice, including consent withdrawal, please see our Privacy Policy.*

***Reviewer #1:*** *No*

***Reviewer #2:*** *No*

**Dear ReviewerS. Thank you very much for spending your time reviewing our manuscript. All your comments are very thoughtful and helpful.**
