# Supplementary material for: Preferences for cesarean section among pregnant women at a tertiary hospital in Ho Chi Minh City, Vietnam: Influencing factors and implications for prenatal care
Source: PLoS One. 2025 Oct 29;20(10):e0335082. doi: 10.1371/journal.pone.0335082 (PMC12571310; doi:10.1371/journal.pone.0335082)
Supplement: Attachment — Submitted filename: Response to Reviewers_31july2025.docx [file pone.0335082.s004.docx]

Dear Editor and Reviewers

***Re: PONE-D-24-48699***

***Preferences for Cesarean Section Among Vietnamese Women: Influencing Factors and Implications for Prenatal Care***

***PLOS ONE***

Thank you for providing thoughtful and valuable feedback on the manuscript. We have responded to the reviewer’s comments below and have changed the text accordingly.

We hope we have satisfactorily addressed all comments and look forward to hearing from you about this submission.

**Reviewers' comments:**

**Reviewer's Responses to Questions**

***Comments to the Author***

*1. If the authors have adequately addressed your comments raised in a previous round of review and you feel that this manuscript is now acceptable for publication, you may indicate that here to bypass the “Comments to the Author” section, enter your conflict of interest statement in the “Confidential to Editor” section, and submit your "Accept" recommendation.*

*Reviewer #2: (No Response)*

*Reviewer #3: (No Response)*

*Reviewer #4: (No Response)*

*Reviewer #5: (No Response)*

**Response**: Thank you.

**2. Is the manuscript technically sound, and do the data support the conclusions?**

*The manuscript must describe a technically sound piece of scientific research with data that supports the conclusions. Experiments must have been conducted rigorously, with appropriate controls, replication, and sample sizes. The conclusions must be drawn appropriately based on the data presented.*

*Reviewer #2: Partly*

*Reviewer #3: Partly*

*Reviewer #4: Partly*

Reviewer #5: Partly

**Response**: Thank you.

**3. Has the statistical analysis been performed appropriately and rigorously?**

*Reviewer #2: Yes*

*Reviewer #3: I Don't Know*

*Reviewer #4: Yes*

*Reviewer #5: Yes*

**Response**: Thank you.

**4. Have the authors made all data underlying the findings in their manuscript fully available?**

*The* [*PLOS Data policy*](http://track.editorialmanager.com/CL0/http:%2F%2Fwww.plosone.org%2Fstatic%2Fpolicies.action%23sharing/1/010f019753949413-81ee269d-51bd-4ed2-90e4-39b326babd8d-000000/tdoRTeiow_Cykp3sYuUsFDMy5qZkrt5r_tO7S9QRzmY=214) *requires authors to make all data underlying the findings described in their manuscript fully available without restriction, with rare exception (please refer to the Data Availability Statement in the manuscript PDF file). The data should be provided as part of the manuscript or its supporting information, or deposited to a public repository. For example, in addition to summary statistics, the data points behind means, medians and variance measures should be available. If there are restrictions on publicly sharing data—e.g. participant privacy or use of data from a third party—those must be specified.*

*Reviewer #2: Yes*

*Reviewer #3: Yes*

*Reviewer #4: Yes*

*Reviewer #5: Yes*

**Response**: Thank you.

**5. Is the manuscript presented in an intelligible fashion and written in standard English?**

*PLOS ONE does not copyedit accepted manuscripts, so the language in submitted articles must be clear, correct, and unambiguous. Any typographical or grammatical errors should be corrected at revision, so please note any specific errors here.*

*Reviewer #2: No*

*Reviewer #3: Yes*

*Reviewer #4: Yes*

*Reviewer #5: Yes*

**Response**: Thank you.

**6. Review Comments to the Author**

*Please use the space provided to explain your answers to the questions above. You may also include additional comments for the author, including concerns about dual publication, research ethics, or publication ethics. (Please upload your review as an attachment if it exceeds 20,000 characters)*

**Reviewer #2**

*Thank you for addressing most of my queries.*

*Re the use of birth mode vs delivery mode. There are still many references to delivery (e.g Table 1 header vaginal delivery). I suggest doing a search of the document for ‘delivery’ or ‘delivering’ and replacing all with birth or birthing.*

**Response**: Thank you. We have replaced all *‘delivery’* with *“birth”* and *“delivering”* with *“birthing”* in the revised manuscript.

***Introduction***

*Page 4 Line 55: Please clarify that you are referring to ‘respiratory issues in the infant’.*

**Response**: Thank you. We have added “in the infant” to clarify “respiratory issues.*”* **(Introduction, paragraph 1, line 58)**

*Page 5, line 118: The authors claim that birth weight and gestation were collected were collected at an antenatal visit. Please correct this since these would only be available after the birth.*

**Response**: Thank you. In our study, all of variables were collected at an antenatal visit when pregnant women had not given birth. Therefore, the weight is an estimated current fetal weight using ultrasound at the time of study participation. Similarly, “gestational age” refers to “gestational age at study participation”. In the revised manuscript, we have clarified this by using “Estimated current fetal weight using ultrasound” and “Gestational age at study participation”. **(Procedures and measurements, lines 130)**

***Table 1***

*I suggest replacing ‘healthy’ pregnancy with the more commonly used term ‘low risk’.*

**Response**: Thank you. We have replaced ‘*healthy’* to *‘low risk’* in Table 2

*Please classify gestation using the standard groupings (preterm <37 weeks, early (37-38 weeks and late term (39+ weeks).*

**Response**: Thank you. We classified the gestational groupings in the previously revised manuscript with 2 groups (36-<39 weeks, 39+ weeks) **(Table 1)**

We used 39 weeks of gestation for cutting point according to the definition of term pregnancy of ACOG which recommended as full term of pregnancy (<https://www.acog.org/clinical/clinical-guidance/committee-opinion/articles/2013/11/definition-of-term-pregnancy>).

*Discussion.*

*The authors highlight the low CS rates in many EU countries. I suggesting adding a comment about the importance of midwifery models of care in these countries and corresponding low rates of perinatal death.*

**Response**: Thank you. We have added a comment *“It notes that EU countries with strong midwifery-led models, such as the Netherlands and Sweden, report lower CS rates and perinatal mortality, possibly due to more continuity of care”* **(Discussion, paragraph 2, line 233-235)**

*Please discuss the small proportion of births of >38 weeks gestation. There is a large literature about the long-term consequences for the infant of preterm and early term birth (37-38 weeks gestation).*

**Response**: Thank you for this important comment. We would like to clarify that all participant data, including gestational age, were collected during prenatal visits, prior to delivery. Therefore, the reported gestational age reflects the gestational age at the time of study participation, not at birth. This distinction is important, as it explains the low proportion of women with gestational age >38 weeks in our dataset. We have now clarified this point in the manuscript to avoid confusion, to state: “*The average gestational age at the time of study participation was 37.6 weeks (SD = 1.2)*”

*The Discussion focuses on the need for improved antenatal education. Please discuss if there is also a need for education of clinicians.*

**Response**: Thank you for your comment. We agree that, in addition to improving antenatal education for pregnant women, there is also a critical requirement for targeted education and training for obstetric clinicians. We have added "*Our findings emphasize the critical role of healthcare providers in creating women’s preferences for cesarean section. As recommendations from clinicians were a powerful predictor of CS preference, interventions should also target clinicians through education, guideline reinforcement, and shared decision-making training to decrease unnecessary CS births.*" **(Discussion, paragraph 1, line 222-226)**

*In the response, the authors stated that the study was insufficiently powered to do subgroup analyses stratified by parity and history of CS. Please add this to the study limitations.*

**Response**: Thanh you. We have added “*Third, the sample size constraints reduced the statistical power of the study, restricting the ability to conduct subgroup analyses (e.g., by cesarean section history or parity) and resulting in wide confidence intervals in regression models, which may affect the precision and robustness of the findings.*” in the Discussion **(Discussion, paragraph 5, line 290-293)**

***Reviewer #3***

*Thank you for the opportunity to review this revised manuscript on a cross-sectional study of factors influencing birth mode preferences among pregnant women at a hospital in Vietnam. The authors have strengthened the manuscript in response to previous review comments, but further clarifications are needed.*

*My main concern with technical soundness of this manuscript is that it is not clear how the study variables were defined or assessed. It is good that the questionnaire is attached as a supplementary file, but each of the variables still need to be clearly defined in the manuscript, with details of assessment and scoring methods. For example, how was ‘healthy gestation’ defined for participants? Does the result in Table 2 mean that the participant had a healthy pregnancy (yes/no) or that they believe that a certain birth mode (vaginal/c-section) is preferable after a healthy pregnancy? As another example, how were data on ‘knowledge of benefits and drawbacks’ categorized into a score of ‘good’ or ‘not good’ (Fig 2)? For the outcome variable, how was ‘preference’ defined, and how does it relate to whether a c-section was actually booked for the participant?*

**Response**: Thank you. We have added *“In our study, we defined* ***healthy or low-risk gestation*** *as pregnancy without any reported discomforts, high-risk medical conditions, or complications affecting the mother or fetus up to the time of the interview. The variable* ***mode of birth preference*** *referred to the participant’s self-reported preferred birth method (vaginal or cesarean birth) in the absence of medical indications or institutional constraints.”* **(Procedures and Measurements, page 5, lines 119-123)**

The result in Table 2 mean that the participant had a healthy pregnancy. We replaced ‘*healthy’* to *‘low risk’* in Table 2 as the 2^nd^ reviewer’s suggestion. **(Table 2, pages 8-9)**

We have also added “*A scoring system was applied, whereby respondents who answered at least 25 out of 30 questions correctly were categorized as having “good knowledge.” In contrast, those with fewer than 25 correct answers were considered to have “not good knowledge.”* **(Procedures and Measurements, page 5, lines 125-128)**

*Please also clarify in the manuscript that the birth weight and gestational age data refer to the previous pregnancy. I was confused about this until reading the response to previous reviewers’ comments.*

**Response**: Thank you. In our study, all of variables were collected at an antenatal visit when pregnant women had not given birth. Therefore, the weight is an estimated current fetal weight using ultrasound at the time of study participation. Similarly, “gestational age” refers to “gestational age at study participation”. In the revised manuscript, we have clarified this by using “Estimated current fetal weight using ultrasound” and “Gestational age at study participation”. **(Procedures and measurements, lines 130)**

*The wide confidence intervals for many of the regression analysis results are a concern, which should be addressed in the Discussion.*

**Response:** Thank you. We have added this limitation in the resived manuscript, to state: “*Third, the sample size constraints reduced the statistical power of the study, restricting the ability to conduct subgroup analyses (e.g., by cesarean section history or parity) and resulting in wide confidence intervals in regression models, which may affect the precision and robustness of the findings.*” **(Discussion, paragraph 5, line 290-293)**

*Some corrections are also needed: Line 154 is not accurate (there were no significant associations between the above noted characteristics, based on Table 1*

**Response**: Thank you. We have added “except for previous birth” to make it more accurate **(Result, line 167)**

*In Figure 2, the scale needs to have even increments to accurately portray the results including the spread of CIs.*

**Response:** Thank you for your insightful comment regarding the scale in Figure 2. We agree that accurately representing the spread of confidence intervals is essential for correct interpretation. However, we intentionally used a logarithmic scale on the x-axis to appropriately visualize the adjusted odds ratios (aORs), which are multiplicative in nature.

Using a log scale allows for symmetric interpretation around the null value of 1. For instance, an OR of 0.5 (halving the odds) and 2.0 (doubling the odds) are equidistant from 1 on a log scale, reflecting their proportional impact. This is a standard practice when displaying ORs or relative risks in forest plots. We have reviewed the figure and ensured that tick marks reflect log-based increments (e.g., 0.25, 0.5, 1, 2, 4, 8, etc.).

| **These are the right ways to present Odds Ratio** | **These are the wrong ways to present Odds ratio** |
| --- | --- |
|  |  |
|  |  |

*Comparisons of c-section rates in the Introduction need to distinguish between national data and site-specific studies. Instead of providing more comparisons in the Discussion, it would be better to compare this study’s finding with the hospital and national rates, to discuss the generalizability of the sample. It should also be noted that this study collected preference data, not c-section rate.*

**Response**: Thank you. We have added *“Compared to the national CS rate of approximately 27.5% reported in UNICEF”* and *“Additionally, the CS rate at Hung Vương Hospital has been increasing by approximately 47.6%”* in the Introduction. **(Introduction, page 3, lines 71-72 and lines 74-75)**

In the Discussion, we have adjusted the phrase *“The CS rate”* by *“the women’s CS preference rate”* or *“The proportion of women who preferred CS”* or *“rate of CS preference”* to clarify our goal. **(Discussion, page 11, lines 218, 227, 228)** We have also added *“Moreover, our rate is slightly higher than one study carried out in a general hospital in Vietnam in 2013 (18.1%).”* to provide more comparison in Vietnam. **(Discussion, line 230-231)**

*Regarding statistical analysis, the methods appear appropriate but it is not clear which variables were retained in the final model after applying backwards elimination. Fig 2 includes aOR results for ‘labour companion’s expectations’ but this variable had p>0.05 in Table 2. It should not have been included in the adjusted model, according to the method described in lines 135-136.*

**Response**: Thank you for your insightful comment regarding the variable selection process in our multivariable model.

We would like to clarify that, variables with a p-value < 0.20 in the univariable analysis (Table 1 and Table 2) were considered for inclusion in the initial multivariable logistic regression model. This threshold was chosen to ensure that potentially important predictors were not prematurely excluded, especially given the exploratory nature of the analysis and the complex interplay of non-medical factors influencing delivery preference.

However, in the final model, statistical significance was assessed using the conventional threshold of p < 0.05. Variables that remained in the final model after the backward elimination process were those that met this significance threshold. We have updated the manuscript to state :*“A backward elimination stepwise approach was used in multiple logistic regression with the initial inclusion of all variables with a p-value below 0.2 in the univariate analyses.”* **(Statistical analysis, line 145-147)**

*The manuscript is generally well written.*

**Response**: Thank you very much for your kind words. This encouraging feedback means a great deal to us and motivates us to continue improving the quality of our current and future work.

*Please clarify line 57: I am not sure what ‘using CS in clinical settings’ means.*

**Response**: Thank you. We have rewritten *“The importance of understanding and using CS in clinical settings” to “underscore the importance of carefully considering when and how cesarean sections should be used in clinical practice, ensuring they are applied based on medical necessity rather than non-medical factors”* to clarify the meaning. **(Introduction, page 3, lines 58-60)**

*For lines 97-100, please include a reference for the first sentence, and delete the second sentence.*

**Response**: Thank you. We have included a reference for the first sentence, and delete the second sentence. **(Setting and participants, paragraph 1, line 101-103)**

*Please provide a reference for the statement about cultural beliefs contributing to rising c-section rates (lines 255-256).*

**Response**: Thank you. We added a reference for the statement about cultural beliefs contributing to rising c-section rates **(Discussion, paragraph 3, line 271-272).**

***Reviewer #4***

*In the method, it said cross sectional study. it means that the data will be from at least 2 different hospital. but, it only show from 1 hospital. so, please revise that the study just conduct for 1 specific hospital.*

**Response**: Thank you. We have revised the manuscript to explicitly state that we conducted our study at one hospital: *“We conducted a cross-sectional study from 19th April 2023 to 04th June 2023 at Hung Vuong Hospital, a public tertiary maternity facility in Ho Chi Minh City, Vietnam”* **(Setting and Participants, page 4, lines 97-98)**

This is also our study limitation. Thus, we included this in the limitation section, to state: “*This study was conducted at a single hospital, which may limit the generalizability of the findings to other regions or healthcare settings in Vietnam.*”

*Also, for Perception and knowledge about birth modes and preference for Cesarean section it show that 92% answer Recommendation of healthcare provider for Cesarean section. It need deeper discussion, how in Vietnam healthcare provider more recommend for cesarean section. It need deeper discussion, how in Vietnam healthcare provider more recommend for cesarean section.*

**Response**: Thank you very much for your comment. As shown in Table 2, 92.7% of participants reported **not** receiving a recommendation from healthcare providers to undergo a cesarean section.

***Reviewer #5***

*Cesarean rates worldwide, including in Vietnam, are high and growing, and this paper explores birthing preferences, in a sample of Vietnamese women. The topic is relevant, as many hospitals seek to de-medicalize childbirth by offering options to ease the stress of the mother and therefore improve mother and child outcomes. The sample is a good size to achieve this aim in a cross-sectional survey, and the data analysis methods are consistent with standard procedure.*

***Abstract***

*Include the current worldwide rate and the rate of CS in Vietnam in the first few sentences.*

**Response:** Thank you. We have added “and in Vietnam, the CS rate reached 34.4% in 2021.” **(Abstract, page 2, lines 29-30)**

***Methods***

***Setting and participants***

*On line 96, it sounds like the hospital may be private, could you confirm? What is the SES of the patients who attend?*

**Response**: Thank you. We have added “public” in the front of the phrase “public tertiary maternity hospital.” **(Setting and participants, page 4, line 98)**

As shown in Table 1, about 60% of participants had a personal monthly income of 20 million VND (~750 USD) or less.

*Line 108- do you know why some declined participating in the study?*

**Response**: Thank you. We would like to clarify that, in accordance with ethical considerations, we did not ask participants to provide a reason if they declined to join the study. However, based on our experience during the recruitment process, we observed that the most common reasons for refusal appeared to be a desire to avoid inconvenience (e.g., time constraints or fatigue during hospital visits) and a reluctance to share personal opinions related to childbirth preferences.

*Line 112- Who recluted the participants, and who delivered the questionnaires?*

**Response**: Thank you. We have clarified this in the revised manuscript "*The trained research assistants recluted the participants and conducted face-to-face interviews …*". **(Procedures and measurements, page 5, line 115-116)**

*Line 115- should say “were evaluated” rather than “was evaluated”*

**Response**: Thank you for your comment about the sentence “*Knowledge about birth modes was evaluated based on 30 questions…*”. We would like to clarify that the subject in this sentence is “knowledge” which is an uncountable and singular noun. Therefore, the use of the singular verb “was evaluated” is grammatically appropriate. **(Procedures and measurements, page 5, line 126)**

*Line 118- you mention the medical record extraction was done on the day of the interview which was during a prenatal visit, but in results you present clinical data from the birth, such as week of gestation at birth, and baby weight. This is very confusing, especially since on Line 147 you talk about the participants as if they are infants. Did you do a later record extraction to gather these data? Or are these previous born children of the multiparous women?*

**Response**: Thank you. In our study, all of variables were collected at an antenatal visit when pregnant women had not given birth. Therefore, the weight is an estimated current fetal weight using ultrasound at the time of study participation. Similarly, “gestational age” refers to “gestational age at study participation”. In the revised manuscript, we have clarified this by using “Estimated current fetal weight using ultrasound” and “Gestational age at study participation”. **(Procedures and measurements, lines 130)**

We have also replaced the word “infant or infants” by the word “prenatal fetus or fetuses” in the result **(Result, page 7, line 159)**

***Results***

*Also, it looks from Table 1 that no characteristics were associated with CS preference aside from previous CS experiences. If this is correct, you want to add a sentence to state that.*

**Response**: Thank you. We have revised the sentence “*There were significant associations between the above information and preference for CS.*” into “ *There were no significant associations between the above characteristics and preference for CS, except for previous birth.*” **(Result, page 7, lines 166-167).**

*Somewhere in the results section, maybe where you list birth data (child characteristics), report how many wanted versus how many ended up having a CS.*

**Response**: Thank you. In our study, all of variables were collected at an antenatal visit when pregnant women had not given birth. Unfortunately, we did not collect birth data.

*Lines 155-160- the ordering of these results are confusing and I had to look back and forth to the table several times to understand that the first two sentences in this paragraph reflect the data in the table (nulliparous women set as reference group). I think this needs to be flushed out in the discussion section, and maybe simplified in the results. It is not necessary to repeat exactly the data in the table, so maybe summarize more succinctly the table data (without the numbers) and keep the sentence comparing past vaginal delivery group to past CS group.*

**Response**: Thank you for your comment. To improve clarity and readability, we have revised this section by separating the content into two distinct paragraphs. The first paragraph now presents key demographic and clinical characteristics of participants, while the second focuses specifically on the associations between previous birth experience and CS preference. We have also added transitional phrases to enhance the logical flow of the second paragraph, as suggested.

Paragraph 1: “*During the study period, a total of 599 … Our analysis revealed that…*”

Paragraph 2: “*In the bivariate analysis, there were no significant associations between the above characteristics and preference for CS, except for previous birth (Table 1). Compared to nulliparous women, those with a previous vaginal birth were less likely to prefer CS (OR = 0.47, 95% CI: 0.27–0.84; p = 0.001). In contrast, women with a history of CS birth were much more likely to prefer CS (OR = 18.67, 95% CI: 10.56–33.02; p < 0.001). When vaginal birth was used as the reference group, women with previous CS birth had significantly higher odds of preferring CS (OR = 39.32, 95% CI: 19.39 - 79.71).*”

*Line 165- should be “among … women WHO preferred CS delivery”. Add the word “who”*

**Response**: Thank you. We have added *“who”* in the sentence: *“Among 162 (27.1%) women who preferred CS birth,”* **(Result, paragraph 2, line 177)**

*Line 178- this part mentions multiple logistic regression, but what variables were in the model? In the Statistical analysis section of the Methods it says you use a back elimination stepwise approach with initial inclusion of all variables with p<.05, but in your Table 1 it looks like that would only be “previous delivery”. Please list variables included in adjusted model.*

**Response**: Thank you for your insightful comment regarding the variable selection process in our multivariable model.

We would like to clarify that, variables with a p-value < 0.20 in the univariable analysis (Table 1 and Table 2) were considered for inclusion in the initial multivariable logistic regression model. This threshold was chosen to ensure that potentially important predictors were not prematurely excluded, especially given the exploratory nature of the analysis and the complex interplay of non-medical factors influencing delivery preference.

However, in the final model, statistical significance was assessed using the conventional threshold of p < 0.05. Variables that remained in the final model after the backward elimination process were those that met this significance threshold. We have updated the manuscript to state :*“A backward elimination stepwise approach was used in multiple logistic regression with the initial inclusion of all variables with a p-value below 0.2 in the univariate analyses.”* **(Statistical analysis, line 145-147)**

*Below I copied and pasted the two similar sentences from the univariate and multivariate analysis. It looks like after adjusting, women with previous CS changed from being less likely to being much more likely to prefer CS. If I follow your methods, you adjusted for “previous delivery”, so using it as a predictor variable doesn’t make sense. Please clarify.*

*“Compared to nulliparous women, those with a previous CS delivery were less likely to prefer CS (OR = 0.47, 156 95% CI: 0.27–0.84; p = 0.001).”*

*“Multiparous women with previous CS were more likely to prefer CS than nulliparous women (adjusted Odds Ratio (aOR) = 41.48, 95% CI 180 17.56 - 97.99).”*

**Response**: Thank you. We have double checked the data and have found a mistake in our result in Table 1 where we mis-labled the group names for “previous delivery” variable. The results in multivariate analysis are correct. We have updated the results and have changed the sentences, to state: *“Compared to nulliparous women, those with a previous* ***CS delivery*** *were less likely to prefer CS (OR = 0.47, 95% CI: 0.27–0.84; p = 0.001). In contrast, women with a history of* ***vaginal delivery*** *were much more likely to prefer CS (OR = 18.67, 95% CI: 10.56–33.02; p < 0.001).”* to *“Compared to nulliparous women, those with a previous* ***vaginal birth*** *were less likely to prefer CS (OR = 0.47, 95% CI: 0.27–0.84; p = 0.001). In contrast, women with a history of* ***CS birth*** *were much more likely to prefer CS (OR = 18.67, 95% CI: 10.56–33.02; p < 0.001).”* **(Result, line 167-170)**

***Discussion***

*If I am understanding the results right, only 7.4% of women discussed birth mode with their provider. That is surprising to me and would be good to take up in the discussion. Also, note in the table this figure is 7.3%. Please make sure all numbers in the table are reflected correctly in the text.*

**Response**: Thank you for your thoughtful observation. We would like to clarify that the figure of 7.3% does not reflect the proportion of women who discussed birth mode with their healthcare providers. In clinical practice in Vietnam, as per standard care protocols, healthcare providers routinely discuss delivery mode options with pregnant women. The 7.3% figure specifically refers to the proportion of participants who reported that their healthcare provider recommended cesarean section (CS), regardless of medical indication. We have revised the manuscript text to clarify this point and avoid potential misinterpretation.: *“A total of 44 women (7.3%) received recommendations from medical professionals for CS birth modes.”* **(Result, line 181-182)**

*There is a document with track changes included at the end of the manuscript. I assume this was an error, but please clarify.*

**Response**: Thank you for pointing this out. We included the version with track changes in accordance with the journal’s submission requirements. It is possible that the file you received was a merged version containing all documents we submitted. The journal’s requirement: *“Please include the following items when submitting your revised manuscript: 1. A rebuttal letter that responds to each point raised by the academic editor and reviewer(s). You should upload this letter as a separate file labeled 'Response to Reviewers'; 2. A marked-up copy of your manuscript that highlights changes made to the original version. You should upload this as a separate file labeled 'Revised Manuscript with Track Changes'; 3. An unmarked version of your revised paper without tracked changes. You should upload this as a separate file labeled 'Manuscript'. It will be deleted after the journal agrees with the final version.”*

*Line 252- you already mentioned this belief, so simply refer back to it rather than repeating it*

**Response**: Thank you. We have deleted the repeating sentence:*“Considerable families opt for elective cesarean sections to ensure their baby is born during an auspicious hour, often based on feng shui advice.”*

*7. PLOS authors have the option to publish the peer review history of their article (*[*what does this mean?*](https://track.editorialmanager.com/CL0/https:%2F%2Fjournals.plos.org%2Fplosone%2Fs%2Feditorial-and-peer-review-process%23loc-peer-review-history/1/010f019753949413-81ee269d-51bd-4ed2-90e4-39b326babd8d-000000/SSaMb1YnuGsryQFckZcuUZyGOwoq_TqPXVqTflLhkzU=214)*). If published, this will include your full peer review and any attached files.*

*If you choose “no”, your identity will remain anonymous but your review may still be made public.*

***Do you want your identity to be public for this peer review?*** *For information about this choice, including consent withdrawal, please see our* [*Privacy Policy*](https://track.editorialmanager.com/CL0/https:%2F%2Fwww.plos.org%2Fprivacy-policy/1/010f019753949413-81ee269d-51bd-4ed2-90e4-39b326babd8d-000000/ZrAHQDgdU0HDvCUdNzrXzk4x2K0AOg19uCAqzdwxBOw=214)*.*

*Reviewer #2: No*

*Reviewer #3: No*

*Reviewer #4: No*

*Reviewer #5: No*

**Dear Reviewers. Thank you very much for spending your time reviewing our manuscript. All your comments are very thoughtful and helpful.**
