# Supplementary material for: Preferences for cesarean section among pregnant women at a tertiary hospital in Ho Chi Minh City, Vietnam: Influencing factors and implications for prenatal care
Source: PLoS One. 2025 Oct 29;20(10):e0335082. doi: 10.1371/journal.pone.0335082 (PMC12571310; doi:10.1371/journal.pone.0335082)
Supplement: Attachment — Submitted filename: v3_Response_To_Reviewer_ 03oct2025.docx [file pone.0335082.s005.docx]

03 October 2025

Dear Editor,

***Re: PONE-D-24-48699***

***Preferences for Cesarean Section Among Vietnamese Women: Influencing Factors and Implications for Prenatal Care***

***PLOS ONE***

Thank you for providing thoughtful and valuable feedback on the manuscript. We have responded to the reviewer’s comments below and have changed the text accordingly.

We hope we have satisfactorily addressed all comments and look forward to hearing from you about this submission.

**Reviewers' comments:**

**Reviewer's Responses to Questions**

***Comments to the Author***

**1. If the authors have adequately addressed your comments raised in a previous round of review and you feel that this manuscript is now acceptable for publication, you may indicate that here to bypass the “Comments to the Author” section, enter your conflict of interest statement in the “Confidential to Editor” section, and submit your "Accept" recommendation.**

*Reviewer #2 (No Response)*

*Reviewer #3: (No Response)*

*Reviewer #4: (No Response)*

*Reviewer #5: All comments have been addressed*

**Response**: Thank you.

**2. Is the manuscript technically sound, and do the data support the conclusions?**

*The manuscript must describe a technically sound piece of scientific research with data that supports the conclusions. Experiments must have been conducted rigorously, with appropriate controls, replication, and sample sizes. The conclusions must be drawn appropriately based on the data presented.*

*Reviewer #2: Partly*

*Reviewer #3: Partly*

*Reviewer #4: Partly*

*Reviewer #5: Yes*

**Response:** Thank you.

**3. Has the statistical analysis been performed appropriately and rigorously?**

*Reviewer #2: Yes*

*Reviewer #3: Yes*

*Reviewer #4: Yes*

*Reviewer #5: Yes*

**Response:** Thank you.

**4. Have the authors made all data underlying the findings in their manuscript fully available?**

*The* [*PLOS Data policy*](http://track.editorialmanager.com/CL0/http:%2F%2Fwww.plosone.org%2Fstatic%2Fpolicies.action%23sharing/1/010f019753949413-81ee269d-51bd-4ed2-90e4-39b326babd8d-000000/tdoRTeiow_Cykp3sYuUsFDMy5qZkrt5r_tO7S9QRzmY=214) *requires authors to make all data underlying the findings described in their manuscript fully available without restriction, with rare exception (please refer to the Data Availability Statement in the manuscript PDF file). The data should be provided as part of the manuscript or its supporting information, or deposited to a public repository. For example, in addition to summary statistics, the data points behind means, medians and variance measures should be available. If there are restrictions on publicly sharing data—e.g. participant privacy or use of data from a third party—those must be specified.*

*Reviewer #2: Yes*

*Reviewer #3: Yes*

*Reviewer #4: Yes*

*Reviewer #5: Yes*

**Response:** Thank you.

**5. Is the manuscript presented in an intelligible fashion and written in standard English?**

*PLOS ONE does not copyedit accepted manuscripts, so the language in submitted articles must be clear, correct, and unambiguous. Any typographical or grammatical errors should be corrected at revision, so please note any specific errors here.*

*Reviewer #2: (No Response)*

*Reviewer #3: Yes*

*Reviewer #4: Yes*

*Reviewer #5: Yes*

**Response:** Thank you.

**6. Review Comments to the Author**

*Please use the space provided to explain your answers to the questions above. You may also include additional comments for the author, including concerns about dual publication, research ethics, or publication ethics. (Please upload your review as an attachment if it exceeds 20,000 characters)*

**Reviewer #2**

*Thank you for addressing the majority of my comments.*

*The authors have clarified that information about fetal size and gestation were collected at the time of study participation. However, the categorisation does not discriminate well since 80% of participants fit into the same group for both measures .*

*I strongly suggest to separate those <37 weeks (i.e preterm at the time) from those at 37-38 weeks.*

**Response:** Thank you. We have re-categorised the participants into those <37 weeks, ≥37- <39 weeks, and ≥39 (preterm at the time of participation) from those at 37–38 weeks. This refinement enables a more apparent distinction between preterm and term pregnancies. **(Result, paragraph 1, page 7, lines 162-163; Table 1, page 7-8)**

*It would also be more informative to use estimated fetal weight percentiles to indicate appropriateness of growth.*

**Response:** Thank you very much for this valuable suggestion. We agree that using estimated fetal weight percentiles would provide a more precise and clinically informative assessment of fetal growth. While we did collect estimated fetal weight in grams, conversion into percentiles would require reference to international or national growth charts (e.g., Hadlock, WHO, INTERGROWTH-21st). As our predefined study objectives and analysis plan focused on women’s preferences for cesarean section and related non-medical factors, categorization by percentiles was not included. We therefore present fetal weight in grams only, which aligns with the scope of our study.

We have included this as one of our limitations which should be addressed in future studies, to state: *“Fourth, although estimated fetal weight was collected, we did not assess estimated fetal weight percentiles, which could better reflect fetal growth and warrant further study”* **(Discussion, paragraph 6, lines 311-312).**

***Discussion***

*Since recommendations from clinicians were such a powerful predictor of CS preference, the importance of measures targeting clinicians (page 11, line 232, marked version), should be reiterated in the Abstract and Conclusions as well.*

**Response:** Thank you. We have added the phrase in the conclusion *“Especially, interventions targeting clinician counselling and practice are crucial to reduce unnecessary cesarean deliveries* **(Conclusion, page 14, lines 324-325).**

In the Abstract, we have added the phrase *“while also emphasizing that clinicians’ recommendations are a powerful driver of women’s preference for cesarean section, highlighting the importance of targeted strategies in clinical practice* **(Abstract, page 2, lines 46-48).**

***Reviewer #3***

*The authors have made important clarifications and corrections to the manuscript in response to previous review comments. Thank you for this.*

*There is still an outstanding issue with the definition of variables. A previous review comment requested the authors to clearly define all included variables, but this was only done for certain variables. It is still unclear what others mean, and the attached study questionnaire does not provide any further details on how the questions were asked or the meaning of the items, especially for Section C. Please clearly define all variables in the Procedures and Measurements section. It is clearest for the reader if this is organized with the outcome variable first, then define the other study variables in the order in which they are presented in the data tables.*

**Response:** Thank you. We have added the paragraph *“The outcome variable was women’s preference for delivery mode (vaginal vs. cesarean birth) in the absence of medical indications. Independent variables included sociodemographic characteristics (e.g., age, residence, education, income, antenatal class attendance), obstetric history and current pregnancy status (e.g., parity, previous delivery mode, gestational age, estimated fetal weight), attitudes and expectations toward childbirth (e.g., fear of childbirth pain, belief in safety of CS, family influence, healthcare provider recommendation), and knowledge of benefits and drawbacks of different birth modes. Detailed definitions of all study variables are provided in the included questionnaire.”*

*We have replaced “The variable mode of birth preference referred to the participant’s self-reported preferred birth method (vaginal or cesarean birth) in the absence of medical indications.” by “We defined the variable mode of birth preference as the participant’s self-reported choice of delivery method.”* **(Procedure and Measurement, paragraph 2, line 122-142)**

*I see also that the Results section does not include data for several items that are in the questionnaire. Please clarify why these were left out, or provide the complete frequency data in Tables 1 and 2.*

**Response:** Thank you for this important comment. Not all questionnaire items were included in the Results because several of them were not directly related to our predefined study objectives, and some involved only a very small number of cases. Presenting such sparse or secondary data might have been misleading and could distract from the key findings. For clarity, we therefore limited Tables 1 and 2 to variables that were relevant and had sufficient frequency for meaningful interpretation. In this submission, we have now included all the definition of all variables.

*Please also ensure that the direction of association is acknowledged when presenting the regression results. Line 36 in the Abstract refers to “predictors” but some of the factors were protective against c-section preference. Lines 220-223 in the Discussion also need to clarify this.*

**Response:** Thank you. We have added in the Abstract *“Factors associated with a higher preference for CS”* before *“multiparous women with previous CS, fear of complications from vaginal birth, perceived safety for the baby, family considerations regarding date of birth, advice from relatives, recommendations from healthcare providers, and exposure to negative experiences of other women”* and the phrase “in contrast” before *“knowledge of the benefits and drawbacks of mode of birth (aOR = 0.47; 95% CI: 0.26 – 0.86) and labor companionship expectations (aOR = 0.45; 95% CI: 0.24 – 0.85) were protective factors, associated with a lower preference for CS.”* **(Abstract, page 2, lines 35-46).**

In the discussion, we have also rewritten the paragrapth like this *“While recommendations from clinicians, family advice, and fear of vaginal birth complications strongly increased CS preference, other factors such as good knowledge about delivery modes and the expectation of labor companionship acted as protective influences, reducing women’s likelihood of preferring CS. Interventions should therefore not only target clinicians through education, guideline reinforcement, and shared decision-making training, but also strengthen the protective factors through enhanced prenatal education, counseling, and supportive care during labor to decrease unnecessary CS births.”* **(Discussion, paragraph 1, page 12, lines 256-264)**

*Line 75 – Please clarify “has been increasing by approximately 47.6%” – is this per year or over some other timeframe? It would be clearer to provide the actual rates instead of % increase, if those data are available.*

**Response:** Thank you. This rate was CS rate in 2017 (2333 CS / 4900 deliveries). We have added *“Additionally, the CS rate at Hung Vuong Hospital was 47.6% (2,333 CS out of 4,900 deliveries) in 2017.”* **(Introduction, paragraph 3, page 4, lines 83-84)**

*Lines 113-116, including Figure 1, should be placed at the beginning of the Results section.*

**Response:** Thank you. We have relocated the sentence “*Among the 661 pregnant women approached and recruited using a convenience sampling technique, 599 agreed to participate and completed the study, resulting in a participation rate of 90.6% (599/661)”* and Fig 1 to the beginning of the Results section. **(Results, page 7, line 175-178)**

*Lines 223-227 – This statement about healthcare providers was added in response to another reviewer’s comments. It is an important point but family influence was also significant, and no other interventions are mentioned until the Conclusion. It would be better to highlight recommendations for both family/maternal education (lines 305-307) and healthcare provider education in the same place.*

**Response:** Thank you. we have also rewritten the paragrapth like this *“While recommendations from clinicians, family advice, and fear of vaginal birth complications strongly increased CS preference, other factors such as good knowledge about delivery modes and the expectation of labor companionship acted as protective influences, reducing women’s likelihood of preferring CS. Interventions should therefore not only target clinicians through education, guideline reinforcement, and shared decision-making training, but also strengthen the protective factors through enhanced prenatal education, counseling, and supportive care during labor to decrease unnecessary CS births.”* **(Discussion, paragraph 1, page 12, lines 256-264)**

*Line 234 – Please reword “It notes” to “It is noteworthy”*

**Response:** Thank you. We have reworded this **(Discussion, paragraph 2, page 12, lines 278).**

*Line 257 – I believe the sentence should end after the word ‘childbirth’, then start a new sentence about the Norwegian study, with its citation.*

**Response:** Thank you. We have replaced *“the comma”* with *“the period”* after the word “childbirth” and started a new sentence, and added the citation of the Norwegian study. **(Discussion, paragraph 3, line 304-306)**

***Reviewer #4***

*Please use the right term if the study only in one hospital (that's not cross sectional studies). remove the word cross sectional studies. it can define as case study in vietnam hospital because the data only in few month in 2023*

**Response:** Thank you. We have now removed the word ‘cross sectional’ as suggested.

***Reviewer #5***

*Thank you for your thorough responses. I have reviewed your revised manuscript and you have addressed all of my concerns*

**Response:** Thank you.

*7. PLOS authors have the option to publish the peer review history of their article (*[*what does this mean?*](https://track.editorialmanager.com/CL0/https:%2F%2Fjournals.plos.org%2Fplosone%2Fs%2Feditorial-and-peer-review-process%23loc-peer-review-history/1/010f019753949413-81ee269d-51bd-4ed2-90e4-39b326babd8d-000000/SSaMb1YnuGsryQFckZcuUZyGOwoq_TqPXVqTflLhkzU=214)*). If published, this will include your full peer review and any attached files.*

*If you choose “no”, your identity will remain anonymous but your review may still be made public.*

***Do you want your identity to be public for this peer review?*** *For information about this choice, including consent withdrawal, please see our* [*Privacy Policy*](https://track.editorialmanager.com/CL0/https:%2F%2Fwww.plos.org%2Fprivacy-policy/1/010f019753949413-81ee269d-51bd-4ed2-90e4-39b326babd8d-000000/ZrAHQDgdU0HDvCUdNzrXzk4x2K0AOg19uCAqzdwxBOw=214)*.*

*Reviewer #2: No*

*Reviewer #3: No*

*Reviewer #4: No*

*Reviewer #5: No*

**Dear Reviewer. Thank you very much for spending your time reviewing our manuscript. All your comments are very thoughtful and helpful.**
